# The challenge and opportunity of gut microbiota‐targeted nanomedicine for colorectal cancer therapy

**DOI:** 10.1002/imt2.213

**Published:** 2024-06-10

**Authors:** Yaohua Wei, Feng Shen, Huidong Song, Ruifang Zhao, Weiyue Feng, Yue Pan, Xiaobo Li, Huanling Yu, Giuseppe Familiari, Michela Relucenti, Michael Aschner, Hanping Shi, Rui Chen, Guangjun Nie, Hanqing Chen

**Affiliations:** ^1^ CAS Key Laboratory for Biomedical Effects of Nanomaterials and Nanosafety, CAS Center of Excellence in Nanoscience, National Center for Nanoscience and Technology Beijing China; ^2^ Center of Materials Science and Optoelectronics Engineering University of Chinese Academy of Sciences Beijing China; ^3^ Department of Gastroenterology and Endoscopy, Xinhua Hospital Shanghai Jiao Tong University School of Medicine Shanghai China; ^4^ Guangzhou Twelfth People's Hospital Guangzhou China; ^5^ CAS Key Laboratory for Biomedical Effects of Nanomaterials and Nanosafety, Institute of High Energy Physics Chinese Academy of Sciences (CAS) Beijing China; ^6^ Guangdong Provincial Key Laboratory of Malignant Tumor Epigenetics and Gene Regulation, Guangdong‐Hong Kong Joint Laboratory for RNA Medicine, Medical Research Center, Sun Yat‐Sen Memorial Hospital Sun Yat‐Sen University Guangzhou China; ^7^ Department of Occupational and Environmental Health, School of Public Health Capital Medical University Beijing China; ^8^ Department of Nutrition & Food Hygiene, School of Public Health Capital Medical University Beijing China; ^9^ Department of Anatomical, Histological, Forensic Medicine and Orthopedic Science Sapienza University of Rome Roma Italia; ^10^ Department of Molecular Pharmacology Albert Einstein College of Medicine Bronx New York State USA; ^11^ Department of Gastrointestinal Surgery and Department of Clinical Nutrition, Beijing Shijitan Hospital Capital Medical University Beijing China; ^12^ School of Public Health Capital Medical University Beijing China; ^13^ Beijing Laboratory of Allergic Diseases Beijing Municipal Education Commission Beijing China

## Abstract

The gut microbiota is an integral component of the colorectal cancer (CRC) microenvironment and is intimately associated with CRC initiation, progression, and therapeutic outcomes. We reviewed recent advancements in utilizing nanotechnology for modulating gut microbiota, discussing strategies and the mechanisms underlying their design. For future nanomedicine design, we propose a 5I principle for individualized nanomedicine in CRC management.

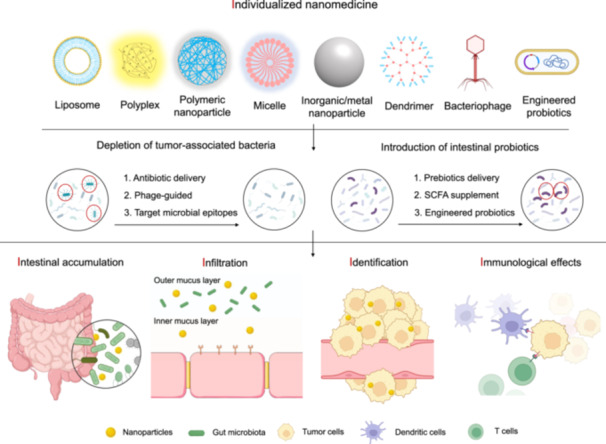

Colorectal cancer (CRC) is the third most prevalent malignancy and has become a clinically challenging disease along with the second leading cause of cancer‐related deaths globally, which is usually attributed to its recurrence and metastasis [1, 2]. Emerging evidence suggests that gut microbiota, especially pathogenic microbes, and their metabolites, constitute an integral component of the tumor microenvironment, demonstrating an intimate association with CRC initiation and progression [1, 2]. While gut microbiota diversity can differ significantly among individuals, large‐scale multicohort analysis of the CRC metagenome has revealed a consistent enrichment of specific microbiomes with tumorigenesis functions, indicating the correlation of microbiota during tumor progression. For example, dysbiotic expansion of intestinal mucosa‐associated *Enterobacteriaceae*, a common pathogen, is strongly associated with inflammatory bowel disease (IBD) progression and CRC occurrence [1, 2, 3]. Moreover, the over‐representation of specific microbiota could potentially counteract traditional treatment regimens and promote tumorigenesis [2]. For instance, *Fusobacterium nucleatum* (*Fn*), a pro‐tumoral bacterium, is rarely found in the lower gastrointestinal tract of healthy individuals but is often enriched in human colorectal cancer (CRC) tumors [4]. Its presence promotes chemoresistance to oxaliplatin and 5‐fluorouracil (5‐FU) in CRC and correlates with poor prognosis in CRC patients [2, 4]. Apart from pro‐tumoral pathogenesis function, some preclinical and clinical studies also underscore the critical role of gut microbiota and its metabolites in improving antitumor responses and therapeutic efficacy of patients with CRC to chemotherapy, radiotherapy, and immunotherapy [2], which is a burgeoning field of gut microbiota‐related interventions. Recent preclinical studies have demonstrated that gut microbiome modulation through oral administration of intestinal commensal bacteria, such as *Bacteroidales*, *Bifidobacterium*, and *Akkermansia muciniphila* (*AKK*), can augment the efficiency of first‐line chemotherapy treatments and enhance the efficacy of CTLA‐4‐ or PD‐L1‐mediated immunotherapies of CRC [1, 2]. Therefore, regulation of gut microbiota and its metabolites, such as antibiotics, fecal microbiota transplants (FMT), probiotics, prebiotics, and diet modifications, is a promising approach to inhibiting colorectal carcinogenesis and improving the therapeutic efficacy of CRC [1, 2]. FMT is the most direct approach for gut microbiota intervention for CRC treatment by reversing gut microbial dysbiosis and ameliorating intestinal inflammation to improve cancer immune responses and anticancer therapeutic efficacy [1, 2]. Supplementations of probiotics and/or prebiotics can inhibit intestinal tumor development and augment their responses to chemotherapy and immunotherapy in patients with CRC [1, 2]. Gut microbiota responds to changes in the intestinal microenvironment by modulating specific bacterial‐derived short‐chain fatty acids (SCFA). SCFA‐guided modulation enhances the anticancer responses and efficacy in mouse and human CRC models [1, 2]. However, current approaches for gut microbiome modulation in treating CRC could not especially manipulate the intratumoral microbiome to enhance the antitumor efficacy. Antibiotic therapy alone lacks tumor‐ and intracellular‐targeting, which limits their effectiveness against tumor‐associated bacteria. Meanwhile, the complexity of gut anatomical physiology and multiple physical barriers impede the precise editing of microbiota through conventional regimes. Recently, nanotechnology has dramatically transformed the landscape of CRC therapy in the past decades [5, 6]. Therefore, there is an urgent need to develop new pathophysiology‐oriented gut microbiota‐targeted therapies in human CRC to precisely remove cancer‐causing pathogenic bacteria or promote gut colonization of beneficial species [7].

## GUT MICROBIOTA‐TARGETED NANOMEDICINE FOR CRC THERAPY

The advantage of nanoscale drug delivery systems lies in their ability to bypass issues related to poor solubility and vulnerability to harsh chemical or biological conditions (such as gastric pH and intestinal enzymes) and in their capacity to enhance the tumor‐targeted accumulation and reduce systemic exposure [5, 6]. The diverse functionality of gut microbiota during CRC progression and treatment provides engineers with multiple facets for precisely modulating the microbiome, enabling the possibility of editing gut microbiota in CRC by applying nanotechnology to employ bacteria‐targeting ligands to guide the drug‐loaded carrier to tumors. These emerging intelligent nanocarriers provide the feasibility of on‐demand gut microbiota modulation (Table 1).

**Table 1:** Representative nanomedicine for gut microbiota modulation in CRC prevention and treatment.

Microbial targeting	Mechanism	Nanomedicine	Drug carriers
Depletion of tumor‐associated bacteria			
*Fusobacterium nucleatum*	Interaction between Fap‐2 and Gal‐GalNAc	Liposomes	Colistin [8]
*Fusobacterium nucleatum*	Phage‐guided targeting	Bioinorganic hybrid bacteriophage	Silver nanoparticles [9]
*Fusobacterium nucleatum*	Phage‐guided targeting	Dextran nanoparticles	Irinotecan [10]
Tumor‐associated bacteria	Electrostatic affinity	Inorganic nanoparticles	TPP [11]
Intracellular microbiome	In response to elevated glutathione	Self‐assembled nanoparticles	MTZ and 5‐FU [12]
Intracellular microbiome	Expose microbial epitopes	Liposomes	Silver and tinidazole [13]
Introduction of intestinal probiotics			
Gut microbiota	Production of anticancer SCFAs	Nanoparticles	Clostridium butyricum and dextran [14]
Gut microbiota	Probiotic proliferation and SCFA production	Xylan‐stearic acid conjugates	Capecitabine [15]
Engineering *Escherichia coli* Nissle 1917	Tumor‐colonizing probiotics	Orally deliverable platform	Heparan sulfate proteoglycan [16]

Abbreviations: 5‐FU, 5‐fluorouracil; MTZ, metronidazole; SCFA, short‐chain fatty acid; TPP, (3‐carboxypropyl) triphenylphosphonium bromide.

The most straightforward strategy for remodeling gut microbiota against CRC development involves directly interfering with the tumor‐colonized or intratumoral microbiome using nanomedicines. The intense interaction between *Fn* membrane protein Fap‐2 and the overexpressed D‐galactose‐β‐(1‐3)‐N‐acetyl‐D‐galactosamine (Gal‐GalNAc) on colorectal tumor cells facilitates *Fn* colonization and significantly reduces immune checkpoint blockade (ICB) efficacy, as evidenced in clinical samples and animal models [8]. Hence, selectively killing the specific microbiome without causing gut dysbiosis is vital during the treatment [9, 10]. Dong and colleagues assembled silver nanoparticles (AgNP) on *Fn*‐binding M13 phage (M13@Ag) to precisely clear *Fn* and remodel the tumor‐immune microenvironment [9]. Oral or intravenous administration of azide‐modified phage nanoparticles to eradicate specific pathobiont significantly counteract the chemoresistance induced by *Fn* and potentiate the activity of folinic acid‐5‐FU‐irinotecan chemotherapy in mouse models of CRC [10]. Recently, a distinct *Fn* clade was reported to dominate CRC progression [17]. Therefore, employing *Fna C2* membrane‐constructed nanomedicine could achieve targeted microbiota eradication without impacting intestinal ecology. Besides *Fn*, other microorganisms like Enterotoxigenic *Bacteroides fragilis* (ETBF) have also been identified in CRC patients and associated with worse prognosis [2]. Therefore, nanomedicines should be designed to eliminate a wide selective range of protumoral pathogens rather than exclusively targeting individual species. Jiang and colleagues proposed a nanocatalytic tumor‐immunotherapeutic method for the selective disintegration of tumor‐associated bacteria (TAB) to trigger innate immunity in situ and cooperatively enhance therapeutic efficacy [11]. Unlike antibiotics, this approach involves a nanocatalytic Fenton reaction under alternating magnetic field‐induced hyperthermia generating cytotoxic hydroxyl radicals in situ. Although the specific *Fn* targeting could be achieved using bacteriophage or bacterial membrane materials, barely any CRC‐associate bacteria‐targeting ligands have been identified. Hence, the main challenge for nanomedicine in modulating CRC‐associated microbiota precisely is achieving efficient delivery of bacteria‐targeting medicines, such as antibiotics, or equipping nanoparticles with newly identified bacteria‐targeting ligands for targeted delivery.

Beyond tumor‐colonized bacteria, increasing evidence supports the existence of an intracellular microbiome in a broad spectrum of tumor cells, disrupting immune surveillance by mediating tumor immune escape and activating immune suppressive pathways in the tumor microenvironment (TME). Therefore, integrating chemotherapeutics with antibiotics represents a synergistic approach for eradicating tumor cells and the intracellular microbiome, offering a strategic advantage in CRC management [12, 18]. Gao and colleagues synthesized an amphiphilic small molecule by combining metronidazole (MTZ) with fluorouridine (5‐FU), which is cleavable in response to elevated glutathione (GSH) levels, enabling the synergistic impact of the released antibiotic and chemotherapeutic, targeting both the intracellular microbiome and tumor cells [18]. In the same way, Chen and colleagues developed an AB‐Gel for tumor‐triggered release of MTZ and 5‐FU, inhibiting CRC cell growth in situ through intestinal perfusion [12]. Moreover, AB‐Gel efficiently suppressed CRC growth and metastasis in an orthotopic mouse model through MTZ‐induced CRC‐related microbiota modulation and enhanced chemotherapy efficacy *via Fn* elimination.

Recent studies propose that intratumoral bacteria, found intracellularly in cancer and immune cells, could offer alternative neoepitopes for cancer immunotherapy. Selectively killing these intracellular bacteria within the tumor may expose microbial epitopes, as alternative sources of cancer‐associated neoantigens. Wang and colleagues engineered liposomes encapsulating an antibiotic silver‐tinidazole complex (LipoAgTNZ) to eradicate tumor‐associated bacteria in both the primary tumor and liver metastases while preserving gut microbiome homeostasis [13]. Mice with CRC colonized with either tumor‐promoting bacteria or probiotics exhibited positive responses to LipoAgTNZ treatment, leading to a long‐term survival rate exceeding 70% in two CRC models infected with *Fn*. Additionally, antibiotic treatment produced microbial neoantigens, activating antitumor CD8^+^ T cells and priming them to recognize infected and uninfected tumors. In summary, nano‐sized antibiotics, whether for intracellular or intertumor bacteria elimination, leverage the advantageous features of nanoparticles, such as enhanced stability, targeting capacity, and improved bioavailability, to advance CRC management. Meanwhile, nanomaterials can be used as promising antibacterial materials for treating gut dysbiosis‐associated diseases, including IBD and CRC, via direct physical interaction with human gut bacteria. These recent discoveries also pave the way for targeted microbiome elimination on demand.

Recently, nanotechnology has been found to improve drug delivery efficiency against abnormally proliferated pathogenic bacteria for CRC therapy through modulation of the intestinal mucosal immune barrier and pathogenic physiological structures [3, 5, 6]. The intestinal barrier is essential in maintaining gut homeostasis and host health [3]. Liu and colleagues presented self‐thermophoretic nanoparticles (CTPB) cloaked in the biomimetic membrane of *Staphylococcus aureus*, aiming for precise localization within the intestinal segment of CRC [19]. Upon near‐infrared laser irradiation, CTPB showed a noteworthy 2.7‐fold enhancement in intestinal mucus penetration efficiency and a simultaneous 3.5‐fold reduction in pathogenic bacterial interception, which significantly enhanced drug delivery efficiency in orthotopic CRC‐bearing mice, along with an impressive 99.4% antitumor rate.

Beyond eliminating pro‐tumoral microbiomes, bioengineered probiotics to colonize CRC tumors selectively enable novel strategies to augment the synergistic effect for CRC prevention and treatment. Probiotic strains have demonstrated safety and have been investigated as biotherapeutic agents for cancer management in preclinical models and human trials. However, current probiotic therapy encounters obstacles related to tumor specificity, unique tumor microenvironment, drug penetration, and dosage adjustability. Engineering tumor‐colonizing *Escherichia coli* Nissle 1917 targeted explicitly to tumor‐targeting adhesion protein heparan sulfate proteoglycan could result in >95% proliferation inhibition of CRC cells and reduce CRC adenoma burden by ~50% in vivo [16]. Engineered lactic acid bacterium *Pediococcus pentosaceus* harboring therapeutic protein P8 could ameliorate impaired gut microbiota and promote CRC regression [20]. Meanwhile, the administered prebiotics could enrich specific bacterial genera and their metabolites, such as SCFAs and cyclic diadenosine monophosphate (cyclic di‐AMP), to potentiate cytotoxic CD8^+^ T cells and active innate immunity, respectively. Zheng and colleagues fabricated prebiotic‐encapsulated probiotic spores (spores‐dex) by utilizing host–guest interactions between commercially available *Clostridium butyricum* and chemically modified dextran. Following oral administration, spores‐dex selectively accumulate in colon cancers, where dextran undergoes fermentation by *Clostridium butyricum*, leading to the production of anticancer SCFAs [14]. Besides, orally administered capecitabine‐loaded prebiotic xylan‐stearic acid nanoparticles significantly improved antitumor immunotherapeutic efficacy from 5.29% to 71.78% and extended the survival time from 14 to 33.5 days in the CRC mouse model by facilitating probiotic proliferation and SCFA production [15].

## A FUTURE PERSPECTIVE ON GUT MICROBIOTA MODULATION IN CRC MANAGEMENT BY INDIVIDUALIZED NANOMEDICINE

**Figure 1:**
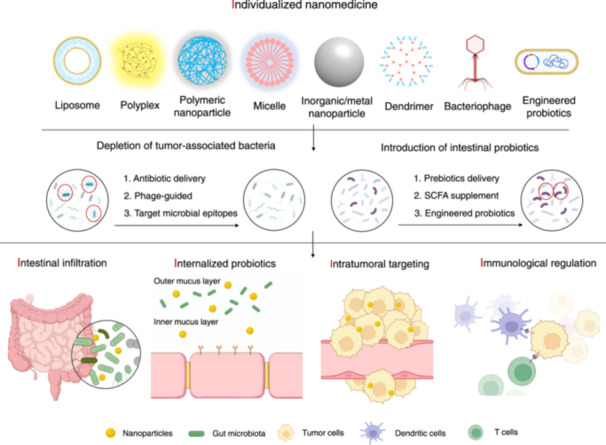
The challenge and opportunity of gut microbiota‐targeted nanomedicine for colorectal cancer (CRC) therapy. A future perspective on gut microbiota‐targeted nanomedicine in CRC prevention and treatment by depleting tumor‐associated pathogens and inducing intestinal probiotics. Antibiotic delivery, phage‐guided nanocarrier, and pathogenic epitope interaction may serve as novel strategies to target and combat tumor‐associated bacteria for CRC therapy. Prebiotics delivery, SCFA supplements, and engineered probiotics may provide an emerging strategy to enhance the potential of colonization of intestinal probiotics and improve CRC prevention and treatment. Meanwhile, the critical challenges of individualized nanomedicine that arise from intestinal infiltration, internalized probiotics, intratumoral targeting, and immunological regulation (5I) will need to be addressed for gut microbiota‐targeted CRC management. Therefore, nanomedicine will usher in a new era of gut microbiota modulation with targeted delivery and stimulus‐responsive payload release, aiming to achieve precise microbiota editing and subsequent CRC therapy.

As opportunities to use nanotechnologies for gut microbiota modulation in CRC prevention and therapy by depletion of tumor‐associated pathogens and induction of intestinal probiotics, critical challenges of individualized nanomedicine that arise from intestinal infiltration, internalized probiotics, intratumoral targeting, and immunological regulation (5I) will need to be addressed for gut microbiota‐targeted in CRC management (Figure 1). (1) Individualized nanomedicine: The tumor microbiome is composed of tumor‐type‐specific intracellular bacteria and is closely associated with CRC progression and the response to anticancer chemotherapy and immunotherapy. Therefore, nanotherapeutic interventions targeting both tumor and gut microbiota should be individualized and applied to the specific case of personalized medicine. (2) Intestinal infiltration: Unlike traditional intravenously infused nanomedicines, which heavily rely on the enhanced permeation and retention (EPR) effect, orally administrable nanomedicines typically face challenges related to low drug bioavailability due to the complex physicochemical and biological barriers of the gastrointestinal tract (GIT). Therefore, future oral nanomedicines should be designed to overcome these barriers and selectively deliver drugs to tumor locations. (3) Internalized probiotics: Probiotic therapy is a safe and effective strategy for CRC prevention and treatment in the future. Engineering probiotics by nanotechnology provides new strategies to reduce the incidence of CRC in patients and improve their quality of life. (4) Intratumoral targeting: Tumor‐associated bacterium is a CRC patient‐unique ecosystem that dynamically changes in response to diet, environment, drugs, and other factors, hence individualized gut microbiota‐targeted nanotherapeutic interventions should be applied to target and kill tumor‐type‐specific and/or patient‐unique microbiome. (5) Immunological regulation: Gut dysbiosis desensitizes the host‐mediated anticancer immune response and results in immunoresistance and poor clinical efficacy; gut microbiota‐targeted nanomedicine provides a promising CRC treatment by combining gut microbiota modulation and immunotherapy.

## CONCLUSION

As previously summarized, major inroads have been achieved in applying nanomedicine for empowered gut microbiota modulation in CRC therapy. Eradicating pathogenic bacteria, supplementing with probiotics or prebiotics, or combining with chemotherapeutics all provide the feasibility of utilizing nanomedicines to fulfill the multifaceted demands during CRC therapy. Therefore, nanomedicine will usher in a new era of gut microbiota modulation with targeted delivery and stimulus‐responsive payload release, aiming to achieve precise microbiota editing and subsequent CRC therapy.

## AUTHOR CONTRIBUTIONS

Hanqing Chen conceived and coordinated this work. Rui Chen and Guangjun Nie provided direction and guidance throughout the preparation of this manuscript. Yaohua Wei, Ruifang Zhao, Huidong Song, and Feng Shen authored the paper. Hanqing Chen revised the manuscript. Yaohua Wei and Hanqing Chen drew the diagrams. Weiyue Feng, Yue Pan, Xiaobo Li, Huanling Yu, Giuseppe Familiari, Michela Relucenti, Michael Aschner, and Hanping Shi revised the paper. All authors have read the final manuscript and approved it for publication.

## CONFLICT OF INTEREST STATEMENT

The authors declare no conflict of interest.

## ETHICS STATEMENT

No animals or humans were involved in this study.

## Data Availability

No new data were generated in this study. Supporting Information (graphical abstract, slides, videos, Chinese translated version, and update materials) may be found in the online DOI or iMeta Science http://www.imeta.science/.
